# A small-molecule TrkB ligand improves dendritic spine phenotypes and atypical behaviors in female Rett syndrome mice

**DOI:** 10.1242/dmm.050612

**Published:** 2024-05-24

**Authors:** Destynie Medeiros, Karen Ayala-Baylon, Hailey Egido-Betancourt, Eric Miller, Christopher Chapleau, Holly Robinson, Mary L. Phillips, Tao Yang, Frank M. Longo, Wei Li, Lucas Pozzo-Miller

**Affiliations:** ^1^ Department of Neurobiology, School of Medicine, University of Alabama at Birmingham, Birmingham, AL 35294, USA; ^2^ Department of Neurology and Neurological Sciences, Stanford University School of Medicine, Stanford, CA 94305, USA

**Keywords:** BDNF, LM22A-4, MeCP2, Pyramidal neuron, Hippocampus

## Abstract

Rett syndrome (RTT) is a neurodevelopmental disorder caused by mutations in *MECP2*, which encodes methyl-CpG-binding protein 2, a transcriptional regulator of many genes, including brain-derived neurotrophic factor (*BDNF*). BDNF levels are lower in multiple brain regions of *Mecp2*-deficient mice, and experimentally increasing BDNF levels improve atypical phenotypes in *Mecp2* mutant mice. Due to the low blood-brain barrier permeability of BDNF itself, we tested the effects of LM22A-4, a brain-penetrant, small-molecule ligand of the BDNF receptor TrkB (encoded by *Ntrk2*), on dendritic spine density and form in hippocampal pyramidal neurons and on behavioral phenotypes in female *Mecp2* heterozygous (HET) mice. A 4-week systemic treatment of *Mecp2* HET mice with LM22A-4 restored spine volume in MeCP2-expressing neurons to wild-type (WT) levels, whereas spine volume in MeCP2-lacking neurons remained comparable to that in neurons from female WT mice. Female *Mecp2* HET mice engaged in aggressive behaviors more than WT mice, the levels of which were reduced to WT levels by the 4-week LM22A-4 treatment. These data provide additional support to the potential usefulness of novel therapies not only for RTT but also to other BDNF-related disorders.

## Teaser

**Summary:** Systemic treatment of female Rett syndrome mice with the brain-penetrant TrkB ligand LM22A-4 restores dendritic spine volume and aggressive behaviors to wild-type levels.

**Research Simplified**

Rett syndrome is a rare neurodevelopmental disorder that mostly affects females. Clinical symptoms of this disorder include intellectual disability, lack of motor control and coordination, irregular breathing, seizures and autistic features. Most individuals with Rett syndrome carry mutations in the X-linked gene that codes for methyl-CpG-binding protein 2 (MeCP2), an important regulator of gene expression and the loss of which is associated with reduced production of another protein, brain-derived neurotrophic factor (BDNF). Understanding exactly how lowered BDNF levels affect Rett symptoms can help researchers develop novel therapeutics for this disorder.

Firstly, the authors used established laboratory female mice lacking the *Mecp2* gene to mimic the key symptoms of Rett syndrome. Because the *Mecp2* gene is located in the X chromosome, the brains of these mice comprise a ‘mosaic’ of neurons, of which some express *Mecp2*, whereas others do not. The new results show that *Mecp2*-expressing neurons have larger dendritic spines compared to those of both neighboring *Mecp2*-lacking neurons as well as *Mecp2*-expressing neurons in unaffected female mice. These observations are important because these spines are the tiny extensions from neurons where information from other neurons arrives and are affected in individuals with Rett syndrome. A 4-week treatment of 5- to 7-month-old female *Mecp2* mutant mice with a BDNF-like molecule, LM22A-4, restored the size of the dendritic spines of *Mecp2*-expressing neurons to their typical size and reduced aggressive behaviours of the mice.

This study uncovered additional therapeutic potential of LM22A-4 for the treatment of Rett syndrome. Further research using improved BDNF-like molecules can help develop rational therapeutics for Rett syndrome.

## INTRODUCTION

Rett syndrome (RTT) is an X chromosome-linked neurodevelopmental disorder associated with intellectual disability and autism that affects approximately 1:10,000 females worldwide (Katz et al., 2012; Neul and Zoghbi, 2004). The majority of individuals with RTT carry loss-of-function mutations in the gene that encodes methyl-CpG-binding protein 2 (MeCP2), a transcriptional regulator that binds to methylated DNA sites and recruits transcriptional repressors and modulates chromatin compaction and its accessibility (Amir et al., 1999; Chahrour et al., 2008; Lyst and Bird, 2015; Nan et al., 1997; Percy and Lane, 2005).

The transcription, synthesis, intracellular transport and activity-dependent release of brain-derived neurotrophic factor (BDNF) are all impaired in a number of neurological disorders, including RTT and Huntington's disease (Gines et al., 2010; Hartmann et al., 2012; Li and Pozzo-Miller, 2014; Tapia-Arancibia et al., 2008). The relevance of BDNF deficiency to RTT pathogenesis is supported by the observations that *Bdnf* expression is directly regulated by MeCP2 in an activity-dependent manner (Chen et al., 2003; Martinowich et al., 2003; Zhou et al., 2006), BDNF levels are lower in multiple brain areas of *Mecp2*-deficient mice (Abuhatzira et al., 2007; Li et al., 2012; Schmid et al., 2012; Wang et al., 2006), and increasing BDNF levels via genetic or pharmacological manipulations improve some of the deficits observed in *Mecp2*-deficient neurons and mice (Chang et al., 2006; Chapleau et al., 2009; Ogier et al., 2007). BDNF signaling via its receptor, tropomyosin-related kinase B (TrkB, encoded by *Ntrk2*[Boxed-text DMM050612B1]), plays a key role in neuronal and synaptic development, as well as in adult synaptic plasticity. BDNF increases the density of dendritic spines of CA1 pyramidal neurons in organotypic cultures of hippocampal slices through TrkB activation of the ERK1/2 pathway (Alonso et al., 2004; Tyler and Pozzo-Miller, 2001). Moreover, hippocampal pyramidal neurons of *Mecp2* knockout (KO) mice have lower dendritic spine density (Chapleau et al., 2012), along with impaired BDNF-induced membrane currents and Ca^2+^ signals mediated by TRPC3 channels (Li et al., 2012), as well as reduced dendritic trafficking and activity-dependent release of BDNF-GFP (Xu et al., 2014). Although these studies indicate that BDNF deficiency is a key component in RTT pathogenesis (Katz, 2014; Li and Pozzo-Miller, 2014), the therapeutic potential of BDNF is limited by its low blood-brain barrier permeability and short plasma half-life (Poduslo and Curran, 1996).

An alternative to BDNF itself is to use synthetic small molecules that target the TrkB receptor as ligands. An established preclinical candidate is LM22A-4, a ‘mimetic’ of the BDNF loop-2 domain that activates TrkB and its downstream signaling pathways (Massa et al., 2010). Indeed, LM22A-4 improved disease phenotypes in mouse models of Huntington's disease (Simmons et al., 2013), Dravet's disease (Gu et al., 2022) and chemotherapy-induced cognitive decline (Geraghty et al., 2019). In female *Mecp2* heterozygous (HET) mice, a 2-month treatment with LM22A-4 improved breathing irregularities (Kron et al., 2012; Schmid et al., 2012), reduced network hyperactivity in hippocampal slices, restored long-term potentiation (LTP) of excitatory synaptic transmission, and improved object location memory (Li et al., 2017). Similarly, a second-generation TrkB ligand based on LM22A-4 also improved breathing patterns and motor deficits in *Mecp2* HET mice (Adams et al., 2020). In the present study, we tested the effects of LM22A-4 on dendritic spine density and size in hippocampal pyramidal neurons of female *Mecp2*-deficient mice, as well as in a machine-learning unbiased screen of open-field behaviors in female *Mecp2* HET mice interacting with unfamiliar and familiar mice.

## RESULTS

### LM22A-4 increases dendritic spine density in pyramidal neurons of hippocampal slice cultures from male *Mecp2* KO mice via activation of TrkB receptors

**Fig. 1.**
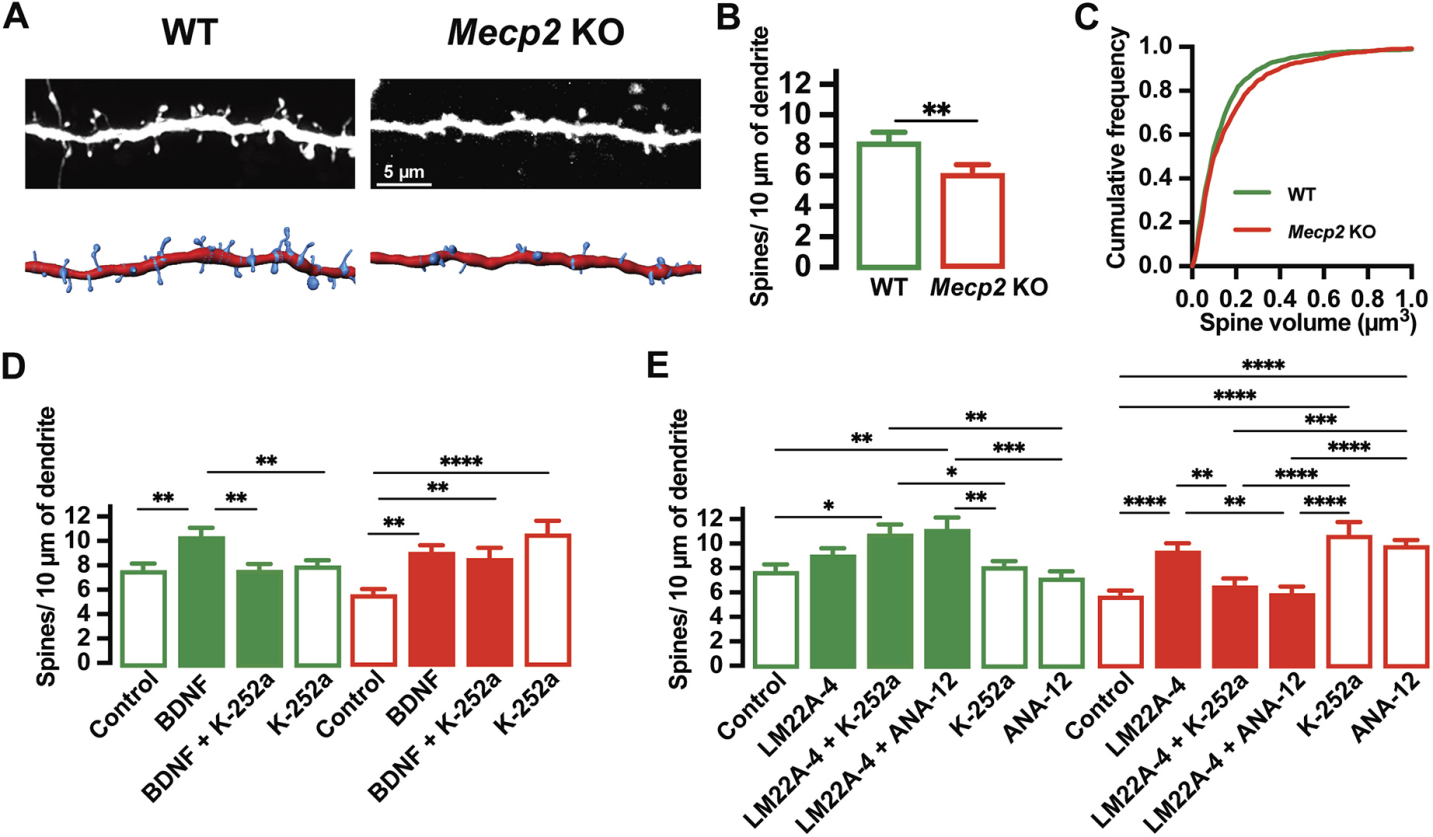
**LM22A-4 increases dendritic spine density in pyramidal neurons of hippocampal slice cultures from male *Mecp2* KO mice via activation of TrkB receptors.** (A) Representative images of eYFP-expressing pyramidal neurons in cultured hippocampal slices from P7 male *Mecp2* KO mice. (B) Cumulative frequency of spine density per micrometer of dendrite in WT and *Mecp2* KO mice. (C) Cumulative frequency of spine volume in WT and *Mecp2* KO neurons. (D) Cumulative frequency of spine density per micrometer of dendrite in WT and *Mecp2* KO mice following treatment with BDNF and the inhibitor K-252a. (E) Cumulative frequency of spine density per micrometer of dendrite in WT and *Mecp2* KO mice following treatment of LM22A-4 and the inhibitors K-252a and ANA-12. Data are mean±s.e.m. *P<0.05; **P<0.01; ***P<0.001; ****P<0.0001 (unpaired two-tailed *t*-test for B; Kolmogorov–Smirnov test for C; one-way ANOVA with Bonferroni's post hoc test for D,E).

We first confirmed that enhanced yellow fluorescent protein (eYFP)-expressing hippocampal CA1 pyramidal neurons in organotypic slice cultures from postnatal day (P) 7 male *Mecp2* KO mice had lower spine density than that of neurons in cultured slices from age-matched male WT mice (*P*=0.0032, unpaired two-tailed *t*-test; *n*=17 *Mecp2* KO neurons/10 slices versus *n*=14 WT neurons/9 slices; Fig. 1A,B), as we reported previously (Chapleau et al., 2012). In addition, the volume of individual spines was larger in CA1 pyramidal neurons in slice cultures from *Mecp2* KO mice [*P*≤0.0001, Kolmogorov–Smirnov (K-S) test; *n*=1458 *Mecp2* KO spines/17 neurons/10 slices versus *n*=2139 WT spines/14 neurons/9 slices; Fig. 1C), as we described previously in dissociated cultures of hippocampal neurons from P1 male *Mecp2* KO mice (Xu et al., 2017) and in CA1 pyramidal neurons in *ex vivo* slices from symptomatic P45-P65 male *Mecp2* KO mice (Li et al., 2016).

Consistent with multiple reports in different experimental preparations, including ours in hippocampal slice cultures from neonatal rats (Alonso et al., 2004; Chapleau et al., 2012, 2008; Tyler and Pozzo-Miller, 2001), BDNF treatment (250 ng/ml, 48 h) increased spine density in CA1 pyramidal neurons in slice cultures from both male *Mecp2* KO mice [*P*=0.0026, one-way ANOVA with Bonferroni's post hoc test (hereafter ANOVA-Bonferroni's); *n*=17 control *Mecp2* KO neurons/10 slices versus *n*=13 BDNF *Mecp2* KO neurons/5 slices] and WT littermates (*P*=0.0042, ANOVA-Bonferroni's; *n*=14 control WT neurons/9 slices versus *n*=15 BDNF WT neurons/5 slices; Fig. 1D). Also in line with prior reports, these effects of BDNF were reduced by treatment with the non-selective Trk receptor inhibitor K-252a (200 nM) in WT slice cultures (*P*=0.0088, ANOVA-Bonferroni's; *n*=13 BDNF+K-252a WT neurons/6 slices versus *n*=15 BDNF WT neurons/5 slices; Fig. 1D). Unexpectedly, K-252a treatment did not alter the effect of BDNF in *Mecp2* KO neurons (*P*>0.9999, ANOVA-Bonferroni's; *n*=15 BDNF+K-252a *Mecp2* KO neurons/6 slices versus *n*=13 BDNF *Mecp2* KO neurons/5 slices) and in fact increased spine density by itself (*P*<0.0001, ANOVA-Bonferroni's; *n*=7 K-252a *Mecp2* KO neurons/4 slices versus *n*=17 control *Mecp2* KO neurons/10 slices; Fig. 1D), which may reflect an altered signaling balance between the low-affinity p75 receptor (encoded by *Ngfr*) and TrkB (Chapleau and Pozzo-Miller, 2012) in the absence of MeCP2.

As expected from its reported activation of TrkB receptors as a partial agonist (Massa et al., 2010) and the lower levels of BDNF in *Mecp2* mutant mice compared to those in WT mice (Katz, 2014; Li and Pozzo-Miller, 2014), LM22A-4 treatment (500 nM, 48 h) increased spine density in pyramidal neurons in areas CA1 and CA3 of hippocampal slice cultures from male *Mecp2* KO mice (*P*<0.0001, ANOVA-Bonferroni's; *n*=17 control *Mecp2* KO neurons/10 slices versus *n*=10 LM22A-4 *Mecp2* KO neurons/7 slices; Fig. 1E). Because of its partial agonism at TrkB receptors (Massa et al., 2010), LM22A-4 had no effect on spine density in pyramidal neurons from male WT mice (*P*>0.9999, ANOVA-Bonferroni's; *n*=12 control WT neurons/11 slices versus *n*=13 LM22A-4 WT neurons/9 slices), where typical BDNF levels outcompete LM22A-4 for TrkB binding. Similar to the effect of BDNF described above, K-252a and the selective TrkB inhibitor ANA-12 (Cazorla et al., 2011) both reduced the effect of LM22A-4 on spine density in *Mecp2* KO neurons (*P*=0.0011, ANOVA-Bonferroni's; *n*=10 LM22A-4+ANA-12 *Mecp2* KO neurons/7 slices versus *n*=10 LM22A-4 *Mecp2* KO neurons/7 slices; Fig. 1E). Similar to the spinogenic effect of K-252a, ANA-12 also increased spine density by itself in *Mecp2* KO neurons (*P*<0.0001, ANOVA-Bonferroni's; *n*=11 ANA-12 *Mecp2* KO neurons/5 slices versus *n*=10 LM22A-4 *Mecp2* KO neurons/7 slices; Fig. 1E).

### LM22A-4 modulates spine density only in MeCP2-expressing CA1 pyramidal neurons in female heterozygous MeCP2-GFP mice

**Fig. 2.**
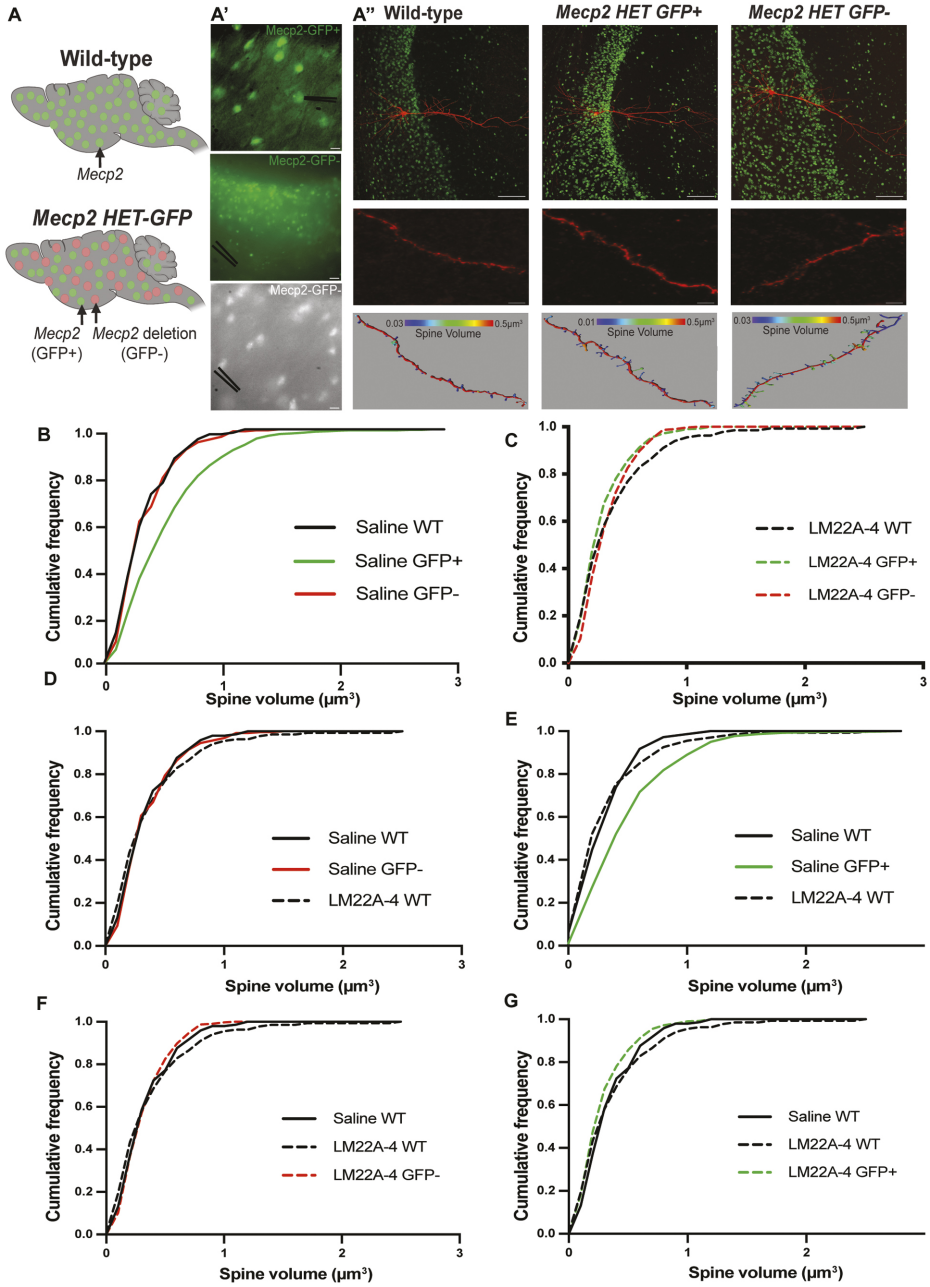
**Systemic LM22A-4 treatment reduces the volume of CA1 dendritic spines of MeCP2-expressing neurons in the mosaic brain of female MeCP2-GFP HET mice without affecting that of MeCP2-lacking neurons.** (A) Representative schematic of a female WT brain with all neurons expressing GFP-tagged MeCP2 protein, and a female MeCP2-GFP HET brain in which *Mecp2-*expressing neurons express GFP-tagged MeCP2 and *Mecp2*-lacking neurons do not. The schematic was created using BioRender.com. (A′) Top: fluorescence image of a MeCP2-GFP-expressing hippocampal CA1 pyramidal neuron in MeCP2-GFP HET mice. Middle: fluorescence image of a MeCP2-GFP-lacking neuron in MeCP2-GFP HET mice. Bottom: differential interference contrast image of a MeCP2-GFP-lacking neuron in MeC2-GFP HET mice. The black lines represent the patch pipette for biocytin cell filling. Scale bars: 10 μm (top, bottom); 30 μm (middle). (A″) Top and middle: representative images of biocytin (red)-filled CA1 pyramidal neurons in slices from female WT and female MeCP2-GFP HET mice. Bottom: representative images of dendritic spines. Scale bars: 100 μm (top); 10 μm (middle). (B-G) Cumulative probability distributions of individual spine volumes in CA1 pyramidal neurons in slices from WT and MeCP2-GFP HET mice treated with LM22A-4 or saline (control).

To identify neurons of known genotypes in the ‘mosaic’ brain of female *Mecp2* HET mice (due to X-chromosome inactivation), we crossed female *Mecp2* HET mice with male mice expressing GFP-tagged MeCP2 (Schmid et al., 2012). For quantitative analyses of dendritic morphology, CA1 pyramidal neurons in *ex vivo* hippocampal slices from young adult mice were identified as MeCP2-expressing or MeCP2-lacking based on the nuclear presence of GFP, and were filled with biocytin through a whole-cell patch pipette, followed by confocal microscopy of Alexa Fluor 488-tagged streptavidin (Fig. 2A).

Mirroring the spine phenotype of symptomatic male *Mecp2* KO mice (P45-P65), in which their density is similar to that in age-matched male WT mice (Li et al., 2016), spine density was similar in the two cellular genotypes in 6-month-old female MeCP2-GFP HET mice, and comparable to that in age-matched female WT mice (*P*>0.9999, ANOVA-Bonferroni's; *n*=35 MeCP2-expressing HET neurons/18 slices, *n*=24 MeCP2-lacking HET neurons/14 slices, *n*=9 WT neurons/6 slices; *n*=4-5 mice, with half receiving LM22A-4; Fig. S1). Unexpectedly, MeCP2-expressing CA1 pyramidal neurons in female MeCP2-GFP HET mice had dendritic spines with larger volumes than those of neighboring MeCP2-lacking neurons and CA1 pyramidal neurons in slices from age-matched female *Mecp2* HET mice (*P*<0.0001, K-S test; *n*=521 spines/35 MeCP2-expressing neurons/18 slices in HET, *n*=257 spines/24 MeCP2-lacking neurons/14 slices in HET, *n*=145 spines/9 neurons/6 slices in WT; Fig. 2B). This observation suggests that dendritic spines of MeCP2-expressing neurons in the mosaic brain of female MeCP2-GFP HET mice retain their sensitivity to neuronal activity (Segal et al., 2000) under the conditions of heightened hippocampal activity observed in female *Mecp2* HET mice (Li et al., 2017), which is comparable to that in the hippocampus of male *Mecp2* KO mice (Calfa et al., 2011, 2015; Li et al., 2016).

Four- to six-month-old female MeCP2-GFP HET mice and their age-matched WT littermates were treated with either LM22A-4 (50 mg/kg in saline, twice daily) or vehicle (saline) for 4 weeks, as described previously (Kron et al., 2012; Li et al., 2017; Schmid et al., 2012). Consistent with the phenotype described above, LM22A-4 did not affect spine density (*P*>0.9999, ANOVA-Bonferroni's, *n*=48 MeCP2-expressing neurons/24 slices in LM22A-4 HET versus *n*=28 MeCP2-lacking neurons/18 slices in LM22A-4 HET; versus *P*=0.0423, K-S test, *n*=11 LM22A-4 WT neurons/9 slices; *n*=4-5 mice, with half receiving LM22A-4; Fig. S1). However, LM22A-4 reduced spine volume only in MeCP2-expressing pyramidal neurons of female MeCP2-GFP HET mice, reaching values comparable to those in MeCP2-lacking neurons in control HET mice (*P*>0.9999, K-S test; *n*=505 spines/48 MeCP2-expressing neurons/24 slices in LM22A-4 HET versus *n*=257 spines/24 MeCP2-lacking neurons/14 slices in control HET) as well as those in female control WT mice (*P*>0.9999, K-S test; *n*=505 spines/48 MeCP2-expressing neurons/24 slices in LM22A-4 HET versus *n*=145 spines/9 neurons/6 slices in WT; Fig. 2C-G).

Similar to its actions *in vitro*, LM22A-4 had no effect on spine volume in neurons of female WT mice (*P*>0.9999, K-S test; *n*=134 spines/11 neurons/9 slices in LM22A-4 WT versus *n*=145 spines/9 neurons/6 slices in control WT). Spine volume was similar in the neurons of control WT mice and LM22A-4-treated WT mice, and MeCP2-lacking neurons in LM22A-4-treated HET mice (*P*>0.9999, K-S test; *n*=145 spines/9 neurons/6 slices in control WT, *n*=134 spines/11 neurons/9 slices in LM22A-4 WT, *n*=303 spines/28 MeCP2-lacking neurons/18 slices in LM22A-4 HET; Fig. 2F). Spine volumes of MeCP2-expressing neurons in LM22A-4-treated HET mice were partially rescued to levels comparable to those of control WT and LM22A-4 WT (*P*<0.0001, K-S test; *n*=505 spines/48 MeCP2-expressing neurons/24 slices in LM22A-4 HET, *n*=134 spines/11 neurons/9 slices in LM22A-4 WT, *n*=145 spines/9 neurons/6 slices in control WT; Fig. 2G).

### Effect of LM22A-4 on social behaviors in female *Mecp2* heterozygous mice

Four- and six-month-old female *Mecp2* HET mice were used to test the effects of a 4-week treatment with LM22A-4 on behaviors relevant to RTT.

#### Standard three-chamber test

**Fig. 3.**
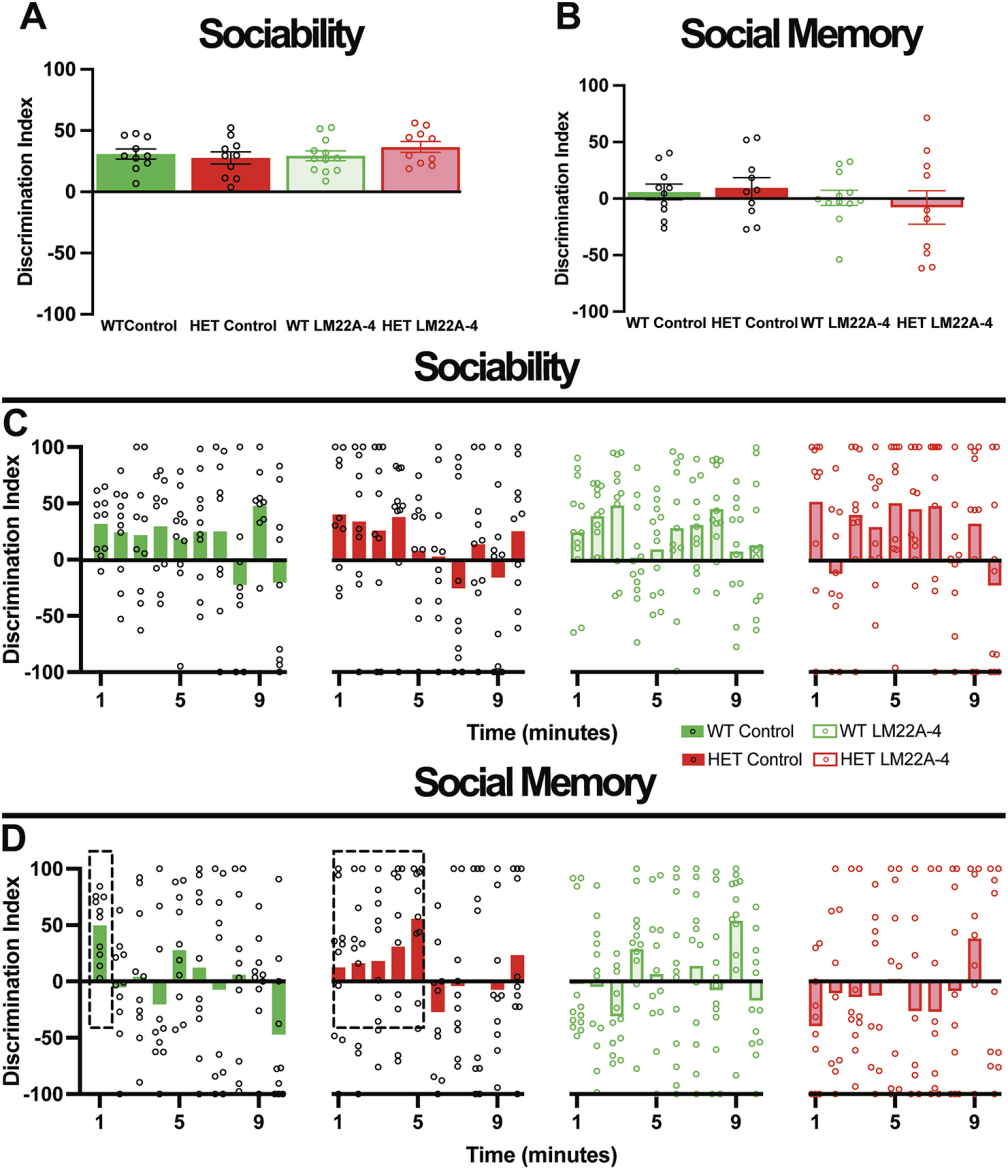
**Female *Mecp2* HET mice have impaired social memory during the first minute of interaction with an unfamiliar mouse, which was not improved by LM22A-4 treatment.** (A,B) Bulk discrimination indices of sociability and memory tests of female *Mecp2* HET mice and WT control littermates, treated with LM22A-4 or vehicle control. (C,D) Minute-by-minute discrimination indices of sociability and memory tests. The boxed data illustrate the time it took for the majority of female *Mecp2* HET mice to achieve a discrimination index comparable to that of female WT mice during the social memory trial. Data are mean±s.e.m.

We used the standard three-chamber social interaction test (Moy et al., 2004) in 7-month-old mice, first for social preference (novel mouse inside an inverted pencil cup versus empty inverted pencil cup), and secondly for social memory [preference for prior novel mouse (familiar) mouse versus new novel (unfamiliar) mouse inside inverted pencil cups], as described previously (Phillips et al., 2019). Using the average interaction time from the whole 10-min session, we found no differences between the discrimination indices for sociability (*P*>0.9999, ANOVA-Bonferroni's; *n*=10 control WT mice versus *n*=10 control *Mecp2* HET mice; Fig. 3A) and social memory (*P*>0.9999, ANOVA-Bonferroni's; control WT versus control *Mecp2* HET mice; Fig. 3B). In line with the absence of differences between genotypes, a 4-week treatment with LM22A-4 did not affect any of these measures either in *Mecp2* HET or in WT mice (*P*>0.9999, ANOVA-Bonferroni's; *n*=10 LM22A-4 *Mecp2* HET mice, *n*=10 control *Mecp2* HET mice, *n*=12 LM22A-4 WT mice, *n*=10 control WT mice; Fig. 3A,B).

Because female mice interact in shorter bouts with their preferred choice mainly at the start of the social preference test compared to males (Netser et al., 2017), the average interaction time from the whole 10-min session may not be the most accurate measure of their behavior. Thus, we compared their discrimination index on a minute-by-minute basis throughout the 10-min session. There were no differences in the sociability trial between female *Mecp2* HET and WT mice (*P*>0.9999, ANOVA-Bonferroni's; control *Mecp2* HET versus control WT; Fig. 3C). However, during the social memory trial, most female WT mice (10 of 10 mice) had a positive discrimination index within the first minute of the test (boxed data in Fig. 3D), suggesting that they could quickly discriminate between a familiar mouse and an unfamiliar mouse. On the contrary, most female *Mecp2* HET mice (8 of 10 mice) took 5 min to reach a discrimination index similar to that of female WT mice (boxed data in Fig. 3D; *P*>0.9999, ANOVA-Bonferroni's; control *Mecp2* HET versus control WT), indicating at least a delayed social memory. After those initial times, neither WT nor *Mecp2* HET mice showed clear socially motivated behaviors (*P*>0.9999, ANOVA-Bonferroni's; control *Mecp2* HET versus control WT; Fig. 3D). Furthermore, LM22A-4 had no effects on these dynamics of social interactions, neither during the sociability test nor on the social memory test (*P*>0.9999, ANOVA-Bonferroni's; among all groups; Fig. 3A-D). These observations are consistent with previous reports of female mice engaging differently during social interactions than males, which engage in longer interaction bouts (Karlsson et al., 2015; Li and Dulac, 2018; Netser et al., 2017; Rodriguez et al., 2023; van den Berg et al., 2015; Williamson et al., 2019).

Seven-month-old female *Mecp2* HET mice covered significantly shorter distances during each of the 10-min sessions of both the sociability phase (empty cup versus novel mouse; *P*=0.0006, ANOVA-Bonferroni's; *n*=9 control WT mice versus *n*=10 control *Mecp2* HET mice; Fig. S2A) and social memory phase (familiar mouse versus unfamiliar mouse; *P*=0.0027, ANOVA-Bonferroni's; *n*=10 control WT mice versus *n*=10 control *Mecp2* HET mice; Fig. S2C) of the three-chamber test. These differences were not altered by LM22A-4 either in the sociability trial (*P*<0.0001, ANOVA-Bonferroni's; *n*=11 LM22A-4 WT mice versus *n*=9 LM22A-4 *Mecp2* HET mice; Fig. S2A) or in the social memory trial (*P*=0.0052, ANOVA-Bonferroni's; *n*=11 LM22A-4 WT mice versus *n*=9 LM22A-4 *Mecp2* HET mice; Fig. S2C). Similarly, 7-month-old female *Mecp2* HET mice exhibited slower velocities than those of age-matched female WT mice in the sociability trial (*P*=0.0006, ANOVA-Bonferroni's; *n*=9 control WT mice versus *n*=10 control *Mecp2* HET mice; Fig. S2B) and in the social memory trial (*P*=0.0028, ANOVA-Bonferroni's; *n*=10 control WT mice versus *n*=10 control *Mecp2* HET mice; Fig. S2D). These differences were also not altered by LM22A-4 either in the sociability trial (*P*<0.0001, ANOVA-Bonferroni's; *n*=11 LM22A-4 WT mice versus *n*=9 LM22A-4 *Mecp2* HET mice; Fig. S2B) or in the social memory trial (*P*=0.0053, ANOVA-Bonferroni's; *n*=11 LM22A-4 WT mice versus *n*=9 LM22A-4 *Mecp2* HET; Fig. S2D). Finally, the total walking times during the 10 min of the unrestricted social assay (see below) were not different between genotypes and were not affected by LM22A-4 (*P*>0.9999, ANOVA-Bonferroni's; among all groups; Fig. 5). This indicates that locomotion deficits may be context dependent and could contribute to the lack of clear sociability and social memory phenotypes in female *Mecp2* HET mice, although female WT mice without this locomotion deficit showed a rather mild preference in the standard three-chamber test, evident only in the first minute (Fig. 3).

#### Unrestricted assay

Because the novel (unfamiliar) and familiar mice used as social stimuli were restrained under inverted pencil cups during the standard social interaction test (Moy et al., 2004), which could result in experimental confounds (e.g. stress vocalizations by stimulus mice), we performed an unbiased assay of naturalistic behaviors shown by a test mouse when interacting with an unfamiliar mouse and a littermate, all freely moving in a large arena (30×40 cm) for 10 min. The three mice were recorded from above, and their individual trajectories were tracked with Mouse Tracker (Motr) software (Ohayon et al., 2013), which was used to train the machine-learning model Janelia Automatic Animal Behavior Annotator (JAABA) (Kabra et al., 2013) with specific behaviors, thus producing an unbiased scoring of behaviors by the test mouse when interacting with freely moving unfamiliar and familiar mice. We successfully used this approach to reveal altered social behaviors in symptomatic male *Mecp2* KO mice (Phillips et al., 2019).

##### Non-aggressive (benign) social interactions

Five-month-old female *Mecp2* HET mice (*n*=6) showed more ‘face-following’ (*P*=0.00358) and more ‘nose-sniffing’ (*P*=0.0005) behavior than that of WT mice (*n*=6, ANOVA-Bonferroni's; Fig. 5A). A 4-week treatment with LM22A-4 reduced nose-sniffing behavior in *Mecp2* HET mice (*n*=6) to levels comparable to those shown by WT mice (*P*=0.0487, ANOVA-Bonferroni's; control *Mecp2* HET versus LM22A-4 *Mecp2* HET), whereas treatment had no effects in WT mice (*n*=9; Fig. 5A).

**Fig. 4.**
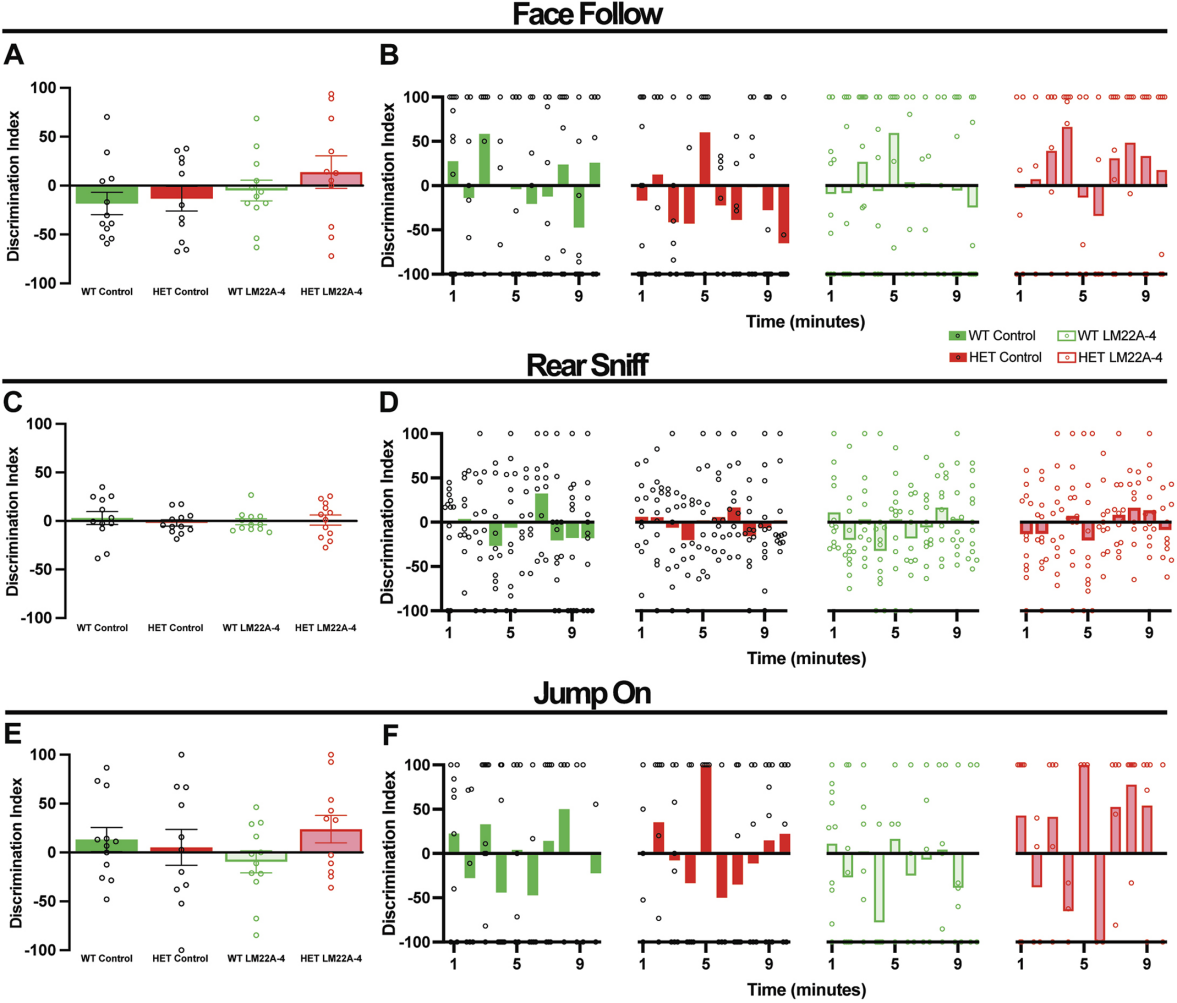
**Social interactions in an unrestricted social assay were not altered in 7-month-old female *Mecp2* HET mice, and were not affected by LM22A-4 treatment.** (A,B) Bulk (A) and minute-by-minute (B) discrimination indices of face-following behavior. (C,D) Bulk (C) and minute-by-minute (D) discrimination indices of rear-sniffing behavior. (E,F) Bulk (E) and minute-by-minute (F) discrimination indices of piggy-back jumping behavior. Data are mean±s.e.m.

**Fig. 5.**
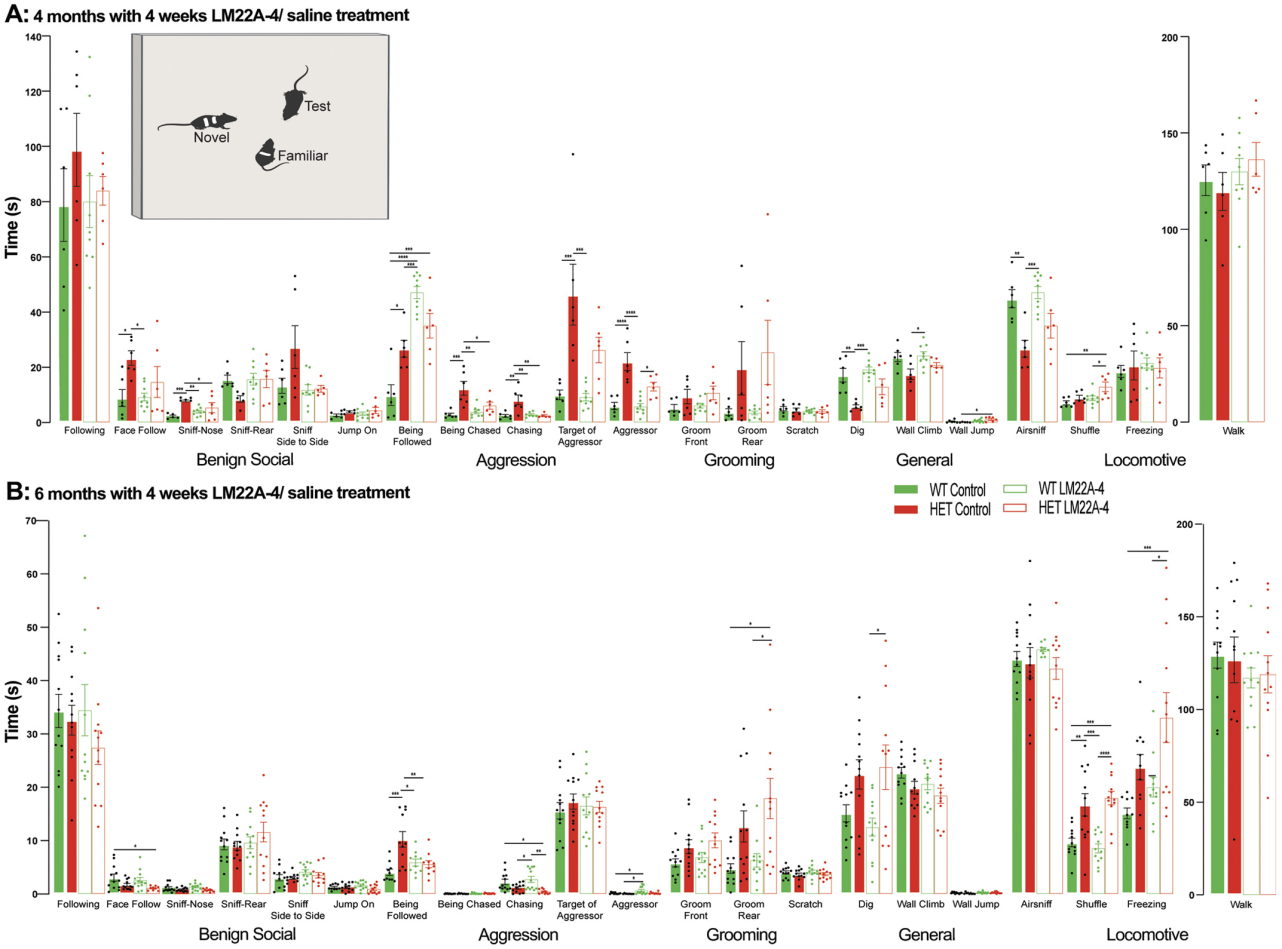
**Unrestricted behavior analysis reveals enhanced aggressive behaviors in *Mecp2* HET mice, which is attenuated by LM22A-4.** Top inset: representative schematic of the unrestricted social assay. The schematic was created using BioRender.com. (A,B) Time spent performing different behaviors using machine-learning scoring of social interactions between three unrestricted female mice: one *Mecp2* HET or WT mouse with one unfamiliar and one familiar female WT mouse. 4-month-old mice (A) or 6-month-old mice (B) were given a 4-week treatment with either LM22A-4 or saline. Data are mean±s.e.m. *P<0.05; **P<0.01; ***P<0.001; ****P<0.0001 (one-way ANOVA with Bonferroni's post hoc test).

In a slightly older cohort of 7-month-old female mice, there were no longer differences in face-following and nose-sniffing behavior (*P*>0.9999, ANOVA-Bonferroni's, *n*=12 *Mecp2* HET and *n*=12 WT mice; Fig. 5B). Furthermore, two other behaviors altered in male *Mecp2* KO mice, ‘rear sniffing’ and atypical ‘piggy-back jumping’ (Phillips et al., 2019) were unchanged between female *Mecp2* HET and WT mice (*P*>0.9999, ANOVA-Bonferroni's; Fig. 5B). The same results were obtained using the average discrimination indices of the whole interaction time or broken down to a minute-by-minute basis (*P*>0.9999, ANOVA-Bonferroni's; among all groups; face following, Fig. 4A,B; rear sniffing, Fig. 4C,D; atypical piggy-back jumping, Fig. 4E,F; nose sniffing, Fig. 5B). Consistent with this lack of differences between genotypes, LM22A-4 had no effects on face-following, rear-sniffing, atypical piggy-back jumping and nose-sniffing behaviors shown by *Mecp2* HET (*n*=12) and WT (*n*=12) mice, either using the 10-min average discrimination indices or in a minute-by-minute basis.

##### Target of social interactions

Five-month-old *Mecp2* HET mice were ‘followed’ more than WT mice (*P*=0.0130, ANOVA-Bonferroni's; control WT versus control *Mecp2* HET; Fig. 5A), which was also observed in 6-month-old mice (*P*=0.0003, ANOVA-Bonferroni's; control WT versus control *Mecp2* HET; Fig. 5B). Unexpectedly, 6-month-old mice *Mecp2* HET mice treated with LM22A-4 showed lower times being followed than those for control *Mecp2* HET mice (*P*=0.0074, ANOVA-Bonferroni's; LM22A-4 *Mecp2* HET versus control *Mecp2* HET; Fig. 5B).

##### Aggressive behaviors

Five-month-old female *Mecp2* HET mice exhibited longer periods of ‘aggressive’ behaviors (*P*<0.0001) and ‘chasing’ behaviors (*P*=0.0008) than those for age-matched WT mice (ANOVA-Bonferroni; control *Mecp2* HET versus control WT; Fig. 5A). A 4-week treatment with LM22A-4 reduced the levels of chasing behaviors in *Mecp2* HET mice to WT levels (*P*=0.0033), but it did not affect aggressive behaviors (*P*=0.0180, ANOVA-Bonferroni; LM22A-4 *Mecp2* HET versus control *Mecp2* HET; Fig. 5A). In contrast, no differences between genotypes nor effects of LM22A-4 on aggressive or chasing behaviors were observed in 7-month-old mice (*P*>0.05, ANOVA-Bonferroni's; Fig. 5B).

##### Target of aggression

Five-month-old female *Mecp2* HET mice were the target of aggressive chasing (*P*=0.0008) and aggressive behaviors (*P*=0.0010) for longer times than those for age-matched WT mice (ANOVA-Bonferroni's; control *Mecp2* HET control versus control WT; Fig. 5A). Interestingly, the 4-week LM22A-4 treatment reduced the duration for which *Mecp2* HET mice were being chased aggressively (*P*=0.0359, ANOVA-Bonferroni's; LM22A-4 *Mecp2* HET versus control *Mecp2* HET; Fig. 5A). However, LM22A-4 did not affect the duration of *Mecp2* HET mice being the target of aggressive behaviors (*P*=0.1242, ANOVA-Bonferroni's; LM22A-4 *Mecp2* HET versus control *Mecp2* HET; Fig. 5A).

##### Locomotion

Seven-month-old female *Mecp2* HET mice showed more ‘shuffling’ locomotion than that of age-matched WT mice (*P*=0.0035, ANOVA-Bonferroni's; control *Mecp2* HET versus control WT; Fig. 5B), but this was not affected by LM22A-4 (*P*<0.0001, ANOVA-Bonferroni's; LM22A-4 *Mecp2* HET versus control *Mecp2* HET; Fig. 5B).

##### General behaviors

Five-month-old female *Mecp2* HET mice showed shorter periods of ‘digging’ (*P*=0.0023) and ‘air-sniffing’ behaviors (*P*=0.0052) than those for WT mice (ANOVA-Bonferroni's; control *Mecp2* HET versus control WT; Fig. 5A), but LM22A-4 did not affect these durations (*P*>0.9999, ANOVA-Bonferroni's; LM22A-4 *Mecp2* HET versus control *Mecp2* HET; Fig. 5B).

## DISCUSSION

In the present study, we tested the *in vitro* and *in vivo* effects of the TrkB partial agonist LM22A-4 on dendritic spine density and volume in hippocampal pyramidal neurons of *Mecp2-*deficient mice, as well as in the classical three-chamber test of social behaviors and a novel machine-learning unbiased screen of open-field behaviors in female *Mecp2* HET mice interacting with unfamiliar and familiar mice.

We first confirmed that LM22A-4 mimics the TrkB-dependent spinogenic effect of BDNF in organotypic slice cultures from neonatal male *Mecp2* KO mice, but not in slice cultures from WT mice due to their typical levels of BDNF that outcompete the partial agonism of LM22A-4 on TrkB receptors. This property of LM22A-4 was described in the original paper (Massa et al., 2010) and in all subsequent reports using different experimental mouse models with reduced BDNF levels, including models of Huntington's disease (Simmons et al., 2013), traumatic brain injury-induced epilepsy (Gu et al., 2018), Dravet's disease (Gu et al., 2022), compulsive alcohol drinking (Warnault et al., 2016), non-arteritic anterior ischemic optic neuropathy (Ali Shariati et al., 2018) and chemotherapy-induced cognitive decline (Geraghty et al., 2019).

In the context of RTT, LM22A-4 also showed therapeutic effects in female *Mecp2* HET mice: it improved breathing irregularities (Kron et al., 2014; Schmid et al., 2012) and mitigated network hyperactivity in hippocampal slices, which restored LTP of CA1 excitatory synaptic transmission in hippocampal slices and improved hippocampal-dependent object location memory (Li et al., 2017). Similarly, a second-generation TrkB ligand derived from LM22A-4 also improved breathing patterns and motor deficits in female *Mecp2* HET mice (Adams et al., 2020). Because these studies identified therapeutic effects of a 1- to 2-month LM22A-4 treatment in 3- to 4-month-old female *Mecp2* HET mice, we followed the same dosage regimen to assess the effects of LM22A-4 effects on dendritic spine phenotypes in hippocampal pyramidal neurons, and on an unbiased screen of atypical behaviors at an age where most RTT-like phenotypes appear in female *Mecp2* HET mice (Samaco et al., 2013), including breathing apneas (Kron et al., 2014; Schmid et al., 2012) and altered hippocampal dependent memory (Li et al., 2017).

Using female MeCP2-GFP HET mice revealed a non-cell-autonomous consequence of *Mecp2* deletion on dendritic spines in the mosaic brain of female RTT mice: CA1 hippocampal pyramidal neurons that express MeCP2 (i.e. WT cells) had larger dendritic spines than neighboring neurons lacking MeCP2 (i.e. mutant cells) and those in female WT mice. We interpret these larger spines to reflect an activity-dependent response of MeCP2-expressing neurons to the atypically heightened hippocampal network activity in female *Mecp2* HET mice (Li et al., 2017). In contrast, MeCP2-lacking neurons may have lost their capacity for dendritic spine plasticity, similar to the saturation of LTP in CA1 pyramidal neurons in male *Mecp2* KO mice, caused by atypically stronger excitatory synapses due to impaired synaptic trafficking of GluA1 (encoded by *Gria1*)-containing AMPA receptors (Li et al., 2016). Note that the expression of TrkB receptors is not affected by *Mecp2* deletion (Li et al., 2012). These observations resemble the non-cell-autonomous consequences of *Mecp2* deletion in the primary motor cortex of female *Mecp2* HET mice, where *Mecp2*-expressing neurons have a lower spine density than that of both *Mecp2*-lacking neurons and neurons in female WT mice (Belichenko et al., 2009). Moreover, the size of the cell body of *Mecp2*-expressing neurons in female *Mecp2* HET mice is smaller than that of neurons in female WT mice (Rietveld et al., 2015; Wither et al., 2013; reviewed in Ribeiro and MacDonald, 2020). Interestingly, LM22A-4 reduced the spine volume in MeCP2-expressing neurons to levels seen in female WT mice, which is consistent with its reduction of hippocampal hyperactivity that allowed typical LTP and restored object-location memory in female *Mecp2* HET mice (Li et al., 2017).

Here, we investigated the effects of a 4-week LM22A-4 treatment on social behaviors in female *Mecp2* HET mice, focusing on key aspects such as sociability, social memory and naturalistic behaviors. In the standard three-chamber social interaction test, no differences in sociability and social memory were found, with LM22A-4 treatment demonstrating no effect. Minute-by-minute analysis of the three-chamber social interaction test revealed a delayed social memory in *Mecp2* HET mice in comparison to that in WT controls, an effect not mitigated by LM22A-4 treatment. The unrestricted assay highlighted the efficacy of LM22A-4 in reducing aggressive behaviors in 5-month-old *Mecp2* HET mice, surmising its positive impact on disease manifestations. However, this effect was more limited in an older cohort (7-month-old mice), suggesting a potential impact of disease progression in *Mecp2* HET mice. Notably, at 7 months of age, *Mecp2* HET mice demonstrated increased shuffling locomotion, further emphasizing the progression of the observed effects. All in all, our study brings to light behavioral alterations in female *Mecp2* HET mice, presenting comprehensive social dynamics across the disease progression. These findings demonstrate the potential therapeutic significance of LM22A-4, providing a promising avenue for investigation in the context of RTT and neurodevelopmental disorders.

The progression of behavioral phenotypes in female *Mecp2* HET mice is not a simple process of all deficits equally worsening with age, resembling the onset and waning of different neuropsychiatric signs in individuals with RTT during the regression, pseudo-stationary and deterioration phases of the disorder (Samaco and Neul, 2011). Our results indicate that female *Mecp2* HET mice display more aggressive behaviors at 5 months of age than age-matched female WT mice, which is absent at 7 months of age. Another example is the anxiety phenotype during the open-field test, which was enhanced in 3-month-old female *Mecp2* HET mice, whereas this difference was absent at 5 months of age and returned later at 7 months (Ribeiro and MacDonald, 2022).

Taken together, these data indicate that social and aggression behaviors may be altered in female *Mecp2* HET mice prior to their shuffling locomotion, and that LM22A-4 only improves some of the affected behaviors. Interestingly, social aggression was reported in male mice lacking *Mecp2* in either *Sim1*-expressing or serotonergic PET1 (FEV)-expressing neurons (Samaco et al., 2009). Similarly, *Mecp2* deletion in PET1-expressing neurons led to an aggressive phenotype in male mice, despite a lack of an anxiety phenotype, as tested in the open-field or ‘light-dark box’ assays (Fyffe et al., 2008). In a different study, male *Mecp2* KO mice showed hyper-reactive escape and defensive behaviors in a ‘mouse defense test battery’ assay (‘predator avoidance’, ‘chase/flight’, ‘closed-door approach’, and ‘forced-contact’ tests), despite the lack of an anxiety phenotype, as tested in the ‘elevated plus’ maze and ‘elevated zero’ maze (Pearson et al., 2015). Intriguingly, a phenotype-based genetic association study revealed that MeCP2 protein levels in mice and *MECP2* single nucleotide polymorphisms in individuals with schizophrenia are associated with social aggression behaviors (Tantra et al., 2014). Furthermore, studies in male mice with deletions or knockdown of either *Bdnf* or *TrkB* display enhanced aggressive phenotypes (Adachi et al., 2017; Ito et al., 2011; Lyons et al., 1999), enhanced anxiety and have increased body weight (Rios et al., 2001). Overall, our results demonstrate that female *Mecp2* HET mice may have altered aggression phenotypes, which should be further explored in deeper detailed in future studies.

Genetic background also contributes significantly to behavioral phenotypes in RTT mice: female *Mecp2* HET mice bred into two different mixes of genetic strains, FVB/N×129S6/SvEv and 129S6/SvEv×C57BL/6, showed altered sociability, contextual fear memory and passive avoidance behaviors at 3 and 5 months of age, whereas at 4 months of age, they showed shorter distance traveled in the open field, suggestive of motor impairment (Samaco et al., 2013). At 5 weeks of age, *Mecp2* HET mice spent more time in the open arm in the elevated plus maze test and greater time in the light side of the light-dark box assay (Samaco et al., 2013). These data are further recapitulated by *Mecp2*^1lox^ mice spending more time in the open arms of the open-field apparatus as well as the open-zero maze at 8 weeks of age (Stearns et al., 2007). All in all, the reduced anxiety phenotype of *Mecp2* HET mice is consistent among multiple studies, whereas aggressive behaviors in *Mecp2* KO males was also found to be a consistent behavioral phenotype, which was at large unexplored in female mice.

Collectively, our observations indicate that sex, genotype and possibly genetic background are highly determinant for the presentation of behavioral phenotypes in MeCP2-based mouse models of RTT, and, therefore, these criteria should be included when defining the outcome measures used for characterizing the longitudinal progression of the disorder and the efficacy of therapeutic approaches.

## MATERIALS AND METHODS

### Mice

Breeding pairs of mice lacking exon 3 of the X chromosome-linked *Mecp2* gene (B6.Cg-*Mecp2*^tm1.1Jae^, ‘Jaenisch’ strain in a pure C57BL/6 background) (Chen et al., 2001) were purchased from the Mutant Mouse Regional Resource Center at the University of California, Davis (stock #000415). A colony was established at the University of Alabama at Birmingham by mating male WT C57BL/6 mice with female *Mecp2* HET mice. Genotyping was performed by PCR of DNA samples from tail clips at weaning age P28. Male hemizygous *Mecp2* mice (i.e. *Mecp2* KO), develop typically until 5-6 weeks of age (P35-P42), when they begin to exhibit RTT-associated motor symptoms, such as hypoactivity, hind limb clasping and reflex impairments (Chen et al., 2001). Male transgenic mice expressing GFP-tagged MeCP2 (Lyst et al., 2013; The Jackson Laboratory stock #014610) were a gift from Dr Adrian Bird (University of Edinburgh, Scotland) and Dr Ben Philpot (University of North Carolina, USA), and were crossed with female *Mecp2* HET mice, which allows identification of the cellular genotype in the female mosaic brains resulting from X-chromosome inactivation. Female *Mecp2* HET mice develop similar RTT-associated symptoms as those observed in male *Mecp2* KO mice, but with a delayed progression, most evidently starting around 2-3 months of life (Samaco et al., 2013). Animals were handled and housed according to the Committee on Laboratory Animal Resources of the National Institutes of Health (NIH). All experimental protocols were annually reviewed and approved by the Institutional Animals Care and Use Committee (IACUC) of the University of Alabama at Birmingham.

### Organotypic slice cultures

Organotypic hippocampal slice cultures were prepared from male postnatal day 5-7 (P5-P7) *Mecp2* KO mice and WT littermates as previously described (Chapleau et al., 2009; Pozzo Miller et al., 1993). Briefly, 500-µm-thick hippocampal slices were cut with a custom-made wire-slicer (Katz, 1987), plated on tissue culture plate inserts (Millicell-CM, Millipore) inside six-well plates with culture medium containing 78% Neurobasal-A medium without phenol red (Invitrogen), 20% heat-inactivated equine serum (Invitrogen), 2% B27 supplement (Invitrogen) and 0.5 mM L-glutamine (Invitrogen), and placed in an incubator at 36°C, 5% CO_2_ and 90% relative humidity. Serum was titrated out over 3 days *in vitro* (DIV), and treatments were performed in serum-free culture media, as previously described (Chapleau et al., 2008).

### Particle-mediated gene transfer

Seven-DIV slice cultures were transfected with a plasmid encoding eYFP by particle-mediated gene transfer using a Helios Gene Gun (Bio-Rad) as described previously (Alonso et al., 2004; Chapleau et al., 2009). Briefly, the eYFP-encoding cDNA plasmid (Clontech) was precipitated onto colloidal gold particles (1.6 µm; Bio-Rad) and coated onto Tefzel tubing. Slices were bombarded with gold particles accelerated by ∼586.25 kPa of helium gas from a distance of 2 cm using a modified gene-gun nozzle with a 2 µm filter. Biolistic transfections were performed 24 h after culture medium was changed to serum-free culture medium with a commercial antibiotic/antimycotic mixture (1:100; penicillin/streptomycin/amphotericin-B; also known as Fungizone, Invitrogen). 24 h after transfection, the culture medium was changed to serum-free medium without antibiotics or antimycotics.

### *In vitro* BDNF and LM22A-4 treatment

Nine-DIV hippocampal slice cultures were randomly assigned to treatment groups with the following drugs dissolved in serum-free medium: recombinant human BDNF (250 ng/ml, Promega); BDNF+K-252a (200 nM, Calbiochem); K-252a alone; LM22A-4 (500 nM; synthesized and validated in the laboratory of F.M.L.); LM22A-4+K-252a; LM22A-4+ANA-12 (100 µM, Sigma-Aldrich); and ANA-12 alone. Culture medium was removed from culture wells and replaced with drug-containing medium; in addition, 50 μl of drug-containing medium was gently applied on top of each slice. All treatments lasted 48 h. Eleven-DIV slice cultures were fixed with 4% paraformaldehyde in 100 mM phosphate buffer overnight at 4°C, washed with 100 mM phosphate-buffered saline (PBS), trimmed from the filter membrane of the cultured inserts, and mounted on glass slides with Vectashield (Vector Laboratories).

### *In vivo* LM22A-4 treatment

Four- to six-month-old female *Mecp2* HET mice and female MeCP2-GFP HET mice and their age-matched female WT littermates received intraperitoneal injections of either sterile LM22A-4 (50 mg/kg) or vehicle (0.9% NaCl) twice daily for 4 weeks, following an established dosing regime with efficacy in other studies using *Mecp2* mutant mice (Kron et al., 2012; Li et al., 2017; Schmid et al., 2012). Mice were randomly assigned to each treatment. LM22A-4 was prepared daily from sterile stocks and dissolved in sterile saline.

### Intracellular loading of fluorescent dye in *ex vivo* hippocampal slices

Mice were deeply anesthetized with a mixture of 100 mg/kg ketamine and 10 mg/kg xylazine, and transcardially perfused with ice-cold ‘cutting’ artificial cerebrospinal fluid (aCSF), containing 87 mM NaCl, 2.5 mM KCl, 0.5 mM CaCl_2_, 7 mM MgCl_2_, 1.25 mM NaH_2_PO_4_, 25 mM NaHCO_3_, 25 mM glucose and 75 mM sucrose, bubbled with 95% O_2_ and 5% CO_2_. The brain was rapidly removed and cut transversely at 300 µm sections using a vibrating blade microtome (VT1200S, Leica Microsystems) in the same ice-cold cutting aCSF. Slices were transferred to normal oxygenated aCSF containing 119 mM NaCl, 2.5 mM KCl, 2.5 mM CaCl_2_, 1.3 mM MgCl_2_, 1.3 mM NaH_2_PO4, 26 mM NaHCO_3_ and 20 mM glucose (with 95% O_2_ and 5% CO_2_) at 32°C for 30 min, and then allowed to recover for 1 h at room temperature before use. Individual slices were transferred to a submerged chamber mounted on a fixed-stage upright microscope (Axioskop FS or AxioExaminer D1, Zeiss) and continuously perfused at room temperature with normal oxygenated aCSF containing 119 mM NaCl, 2.5 mM KCl, 2.5 mM CaCl_2_, 1.3 mM MgCl_2_, 1.3 mM NaH_2_PO4, 26 mM NaHCO_3_ and 20 mM glucose (with 95% O_2_ and 5% CO_2_). Pyramidal neurons in CA1 stratum pyramidale were visualized by infrared differential interference contrast microscopy with water-immersion objectives (63×0.9 NA, or 20×1.0 NA plus 0.50-4× zoom, Zeiss). In MeCP2-GFP mice, GFP was imaged with 475 nm LED illumination (X-Cite Turbo, Excelitas), a GFP cube (Semrock) and a QuantEM:512SC cooled CCD (Photometrics). Whole-cell pipettes contained 120 mM Cs-gluconate, 17.5 mM CsCl, 10 mM Na-HEPES, 4 mM Mg-ATP, 0.4 mM Na-GTP, 10 mM Na_2_-creatine phosphate, 0.2 mM Na-EGTA and 8 mM biocytin (Sigma-Aldrich), at 290-300 mOsm, pH 7.3 (final resistance, 3-4 MΩ). After ∼15 min of whole-cell access to allow biocytin loading, slices were fixed in 4% paraformaldehyde in 100 mM PBS and stained with streptavidin-conjugated Alexa Fluor 488 (Life Technologies) as described previously (Li et al., 2016).

### Confocal microscopy and dendritic spine analyses

eYFP-expressing CA1 and CA3 pyramidal neurons in hippocampal slice cultures from male *Mecp2* KO mice and male WT littermates, and biocytin-filled, Alexa Fluor 488-labeled CA1 pyramidal neurons in hippocampal slices from female MeCP2-GFP HET mice and female WT littermates were selected for confocal imaging if they showed fluorescent labels throughout the entire dendritic tree and lacked signs of degeneration (e.g. dendritic ‘blebbing’). High-resolution *z*-stack images of apical secondary and tertiary dendrites were acquired with oil-immersion 60× (NA 1.42) objectives (PlanApo) plus 3× digital zoom in either a Fluoview FV-300 (Olympus) or a LSM-800 (Zeiss) confocal microscope. Image stacks were acquired at 0.1 µm intervals in the *z*-plane.

*Z*-stack image stacks were three-dimensionally reconstructed, surface rendered and analyzed semi-automatically with the ‘Filament Tracing’ module of Imaris (Bitplane) (Swanger et al., 2011). Image stacks were loaded into Imaris, a region of interest including a 40-to 80-µm-long dendritic shaft was selected, and ‘Filament Tracing’ was used to trace and render dendrites and their associated spines. Spines were defined as dendritic protrusions shorter than 3 μm. The numerical densities of spines were calculated for each dendritic segment and normalized to 10 μm of dendritic length. The relative intensity of individual spines and their parent dendritic shafts was used to estimate the volume of individual spines (Benavides-Piccione et al., 2013). An experimenter masked to genotype and treatment performed dendritic spine analyses.

### Behavioral analyses

All handling and testing were done in the dark phase of the standard 12 h light/12 h dark cycle (06:00 ON, 18:00 OFF), with the experimenter wearing a red headlamp and infrared illumination for digital videography.

#### Standard three-chamber social test

We used the standard three-chamber social interaction test (Moy et al., 2004). Prior to testing, mice underwent a 3-day acclimation period during which they were familiarized to the experimenter with handling for 3 min each day at the same time as testing. The testing environment was a three-chambered box that contained two empty inverted pencil cups placed in the two side chambers. Mice were placed in the central chamber and allowed to freely explore for 5 min. After this initial acclimation period, mice were directed to return to the central chamber, and barriers were positioned over the side openings. At this time, a novel mouse was placed beneath one of the previously empty pencil cups in one of the two side chambers, alternating between trials. The barriers were then removed and the test mouse was allowed to freely explore the chambers for 10 min. Subsequently, the test mouse was again directed to return to the central chamber and barriers were positioned over the side openings. A second novel mouse was placed beneath the formerly empty pencil cup, the previously added mouse was now considered ‘familiar’. After removing the side barriers, the test mouse was allowed to explore the chambers for an additional 10 min. Following each test, the apparatus was meticulously cleaned with 70% isopropanol. The time that the test mouse spent actively sniffing each pencil cup, containing either a social conspecific or empty, was quantified. For the sociability test, the discrimination index was calculated as: [(time investigating cup with mouse)−(time investigating empty cup)]/[(time investigating cup with mouse)+(time investigating empty cup)]×100. For the social memory trial, the discrimination index was calculated as: [(time investigating cup with unfamiliar mouse)−(time investigating cup with familiar mouse)]/[(time investigating cup with unfamiliar mouse)+(time investigating cup with familiar mouse cup)]×100. Test mice that spent over 75% of the acclimation time in a single compartment were excluded from the study. Statistical analyses were performed on the discrimination indices for each test mouse.

#### Unrestricted assay

One week prior to testing, the backs of sentinel novel and familiar mice were dyed with blond hair dye (Born Blonde Maxi, Clairol) with different patterns for computer vision tracking. ‘Sentinel’ mice were the familiar and unfamiliar mice used as social stimuli for the ‘test’ mouse in the unrestricted social assay. Mice were habituated to the testing arena, an open-field box (30×40 cm) containing clean bedding, for 3 days prior to the testing day, 10 min per day. During the testing day, test mice were habituated to the testing arena for 10 min. Sentinel mice, including one cage-mate and one unfamiliar mouse from a different cage, were placed in the testing arena and were allowed to interact freely for 10 min. After this time, sentinel mice were placed into a neutral cage and the test mouse was returned to their home cage. The arena was meticulously cleaned with 70% isopropanol and filled with new bedding between each test. Sentinel mice interacted with a maximum of five test mice and were no longer used within the study if they fought with other sentinels or displayed excessive grooming phenotypes. Test videos were loading into Motr (https://github.com/motr/motr) (Ohayon et al., 2013) to create tracks that were exported to the machine-learning model JAABA (https://github.com/kristinbranson/JAABA) (Kabra et al., 2013) for unbiased behavioral scoring. *JAABA* classifiers were first trained on pilot data sets. Behavioral scores for social memory and other behaviors were taken from the entire video, as different behaviors emerged at later times during the 10-min trial, and social behavior times were pooled between those for unfamiliar and cage-mate mice. Mice not interacting with sentinels for more than 3 s (out of 240 s) were excluded from analyses. Statistical tests were performed on the time per behavior for each test mouse.

### Statistical analyses

All experiments were repeated in at least three independent slice cultures or *ex vivo* acute slice preparations. All statistical analyses were performed using Prism (GraphPad Software), *P*<0.05 was considered significant. Outliers were detected using the ROUT method. Data are presented as mean±standard error of the mean (s.e.m.) and were compared using unpaired two-tailed Student's *t*-tests for two groups or one-way ANOVA with Bonferroni's post hoc test for more than two groups. Data with more than three groups that did not fit the normal distribution were analyzed using the Kruskal–Wallis test, with Dunn's post hoc test for multiple comparisons; post hoc tests were only performed for ANOVA and Kruskal–Wallis tests that reported *P*<0.05. The Kolmogorov–Smirnov (K-S) test was used for comparisons of cumulative frequency distributions.

## Supplementary Material

10.1242/dmm.050612_sup1 Supplementary information
