# Supplementary material for: A small-molecule TrkB ligand improves dendritic spine phenotypes and atypical behaviors in female Rett syndrome mice
Source: Dis Model Mech. 2024 May 24;17(6):dmm050612. doi: 10.1242/dmm.050612 (PMC11139040; doi:10.1242/dmm.050612)
Supplement: Supplementary information [file dmm-17-050612-s1.pdf]

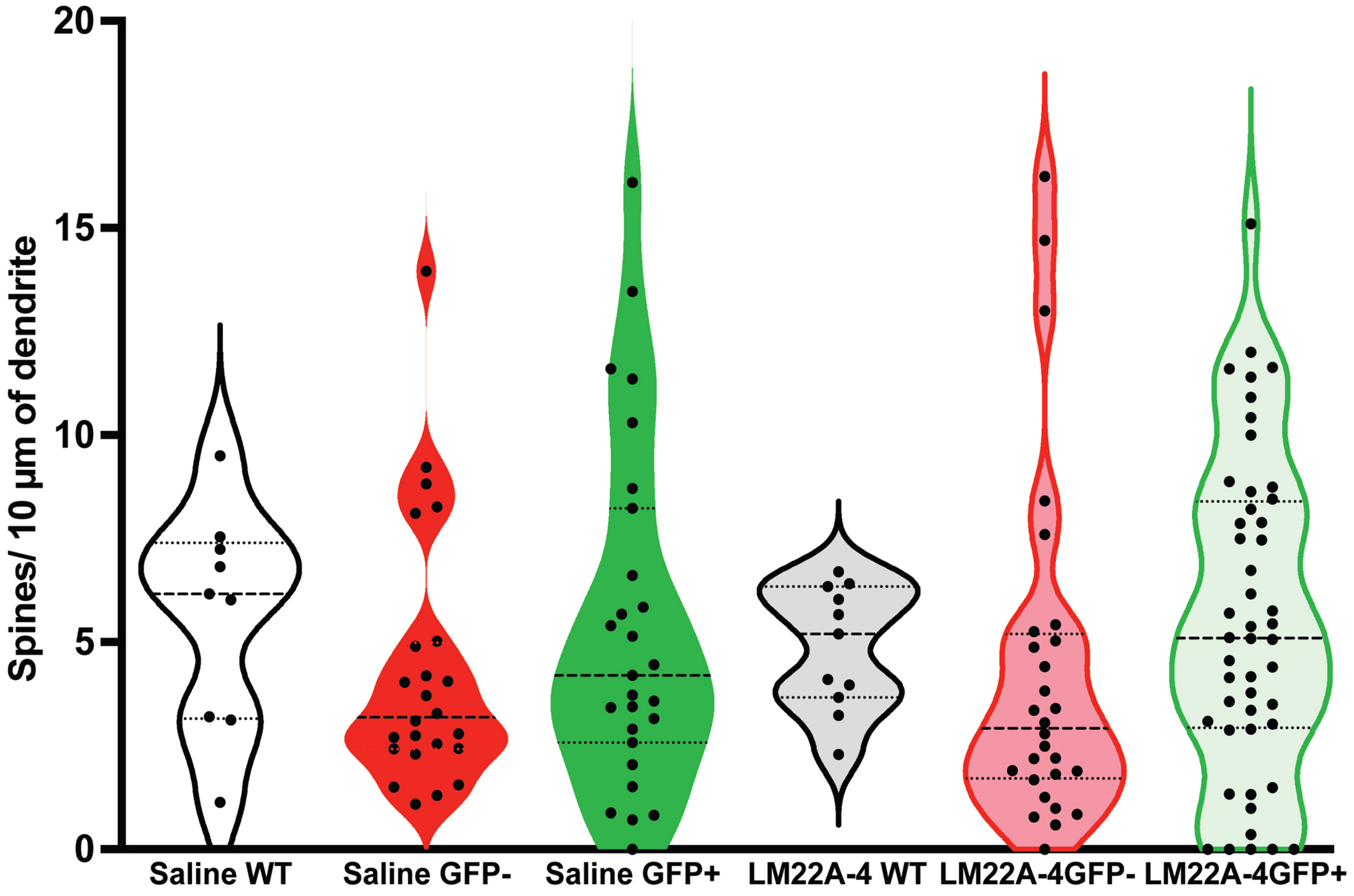

Fig. S1. Dendritic spine density is similar in the two cellular genotypes of female MeCP2-GFP HET mice, and comparable to that in female WT mice.

Cumulative probability distribution of spine density per 10µm of dendrite in WT and MeCP2-HET-GFP expressing or lacking neurons treated with control or LM22A-4. Data are mean ±SEM.

Disease Models & Mechanisms • Supplementary information

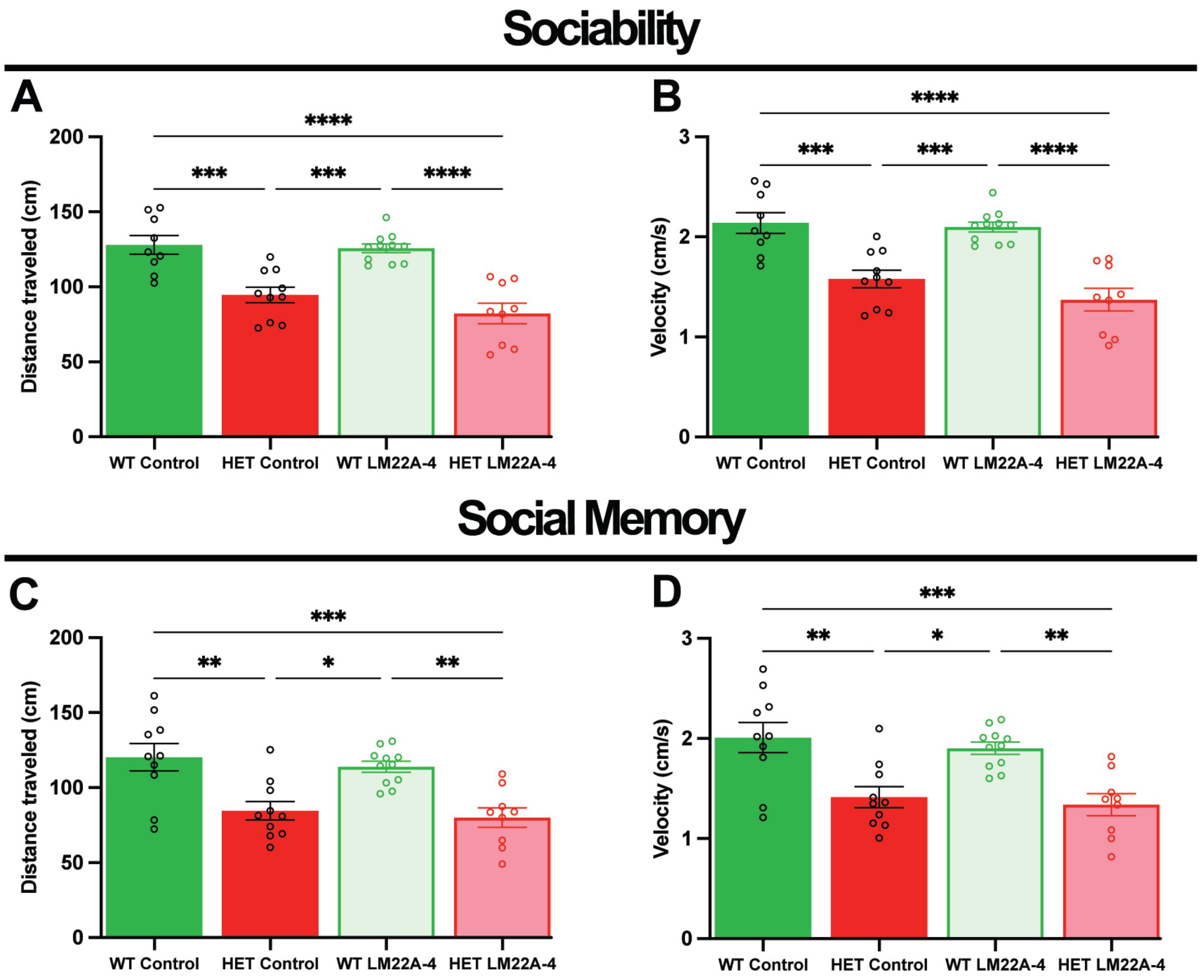

Fig. S2. 7-month old *Mecp2* HET mice have reduced distance traveled and velocity in the 3-chamber social interaction test, LM22A-4 treatment had no effect.

(A) Distance traveled during the sociability trial. (B) Velocity during the sociability trial. (C) Distance traveled during the social memory trial. (D) Velocity during the social memory trial. Data are mean ±SEM. *P<0 05, **P<0.01, *** P<0.001, **** P<0.0001.

Disease Models & Mechanisms • Supplementary information
